# Why a Comprehensive Understanding of Mental Workload through the Measurement of Neurovascular Coupling Is a Key Issue for Neuroergonomics?

**DOI:** 10.3389/fnhum.2016.00250

**Published:** 2016-05-31

**Authors:** Kevin Mandrick, Zarrin Chua, Mickaël Causse, Stéphane Perrey, Frédéric Dehais

**Affiliations:** ^1^Département Conception et conduite des véhicules Aéronautiques et Spatiaux, Institut Supérieur de l'Aéronautique et de l'EspaceToulouse, France; ^2^EuroMov, University of MontpellierMontpellier, France

**Keywords:** mental workload, mental resources, neurovascular coupling, neuroergonomics, electroencephalography, near-infrared spectroscopy

Raja Parasuraman, the father of *Neuroergonomics* (the crossroads of Ergonomics and Neuroscience, Figure 1) has opened the doors to new discoveries and techniques for advancing understanding of human behavior with the underlying brain mechanisms (Parasuraman, 1998). As of his death in 2015, a precise and objective definition of the concept of mental workload (MWL) had still not yet been formulated. In this opinion piece, we posit that MWL is associated through the measurement of neurovascular coupling (NVC); innovative neuroimaging methods is now capable of measuring such a phenomenon; all while highlighting Parasuraman's many contributions to this field.

**Figure 1:**
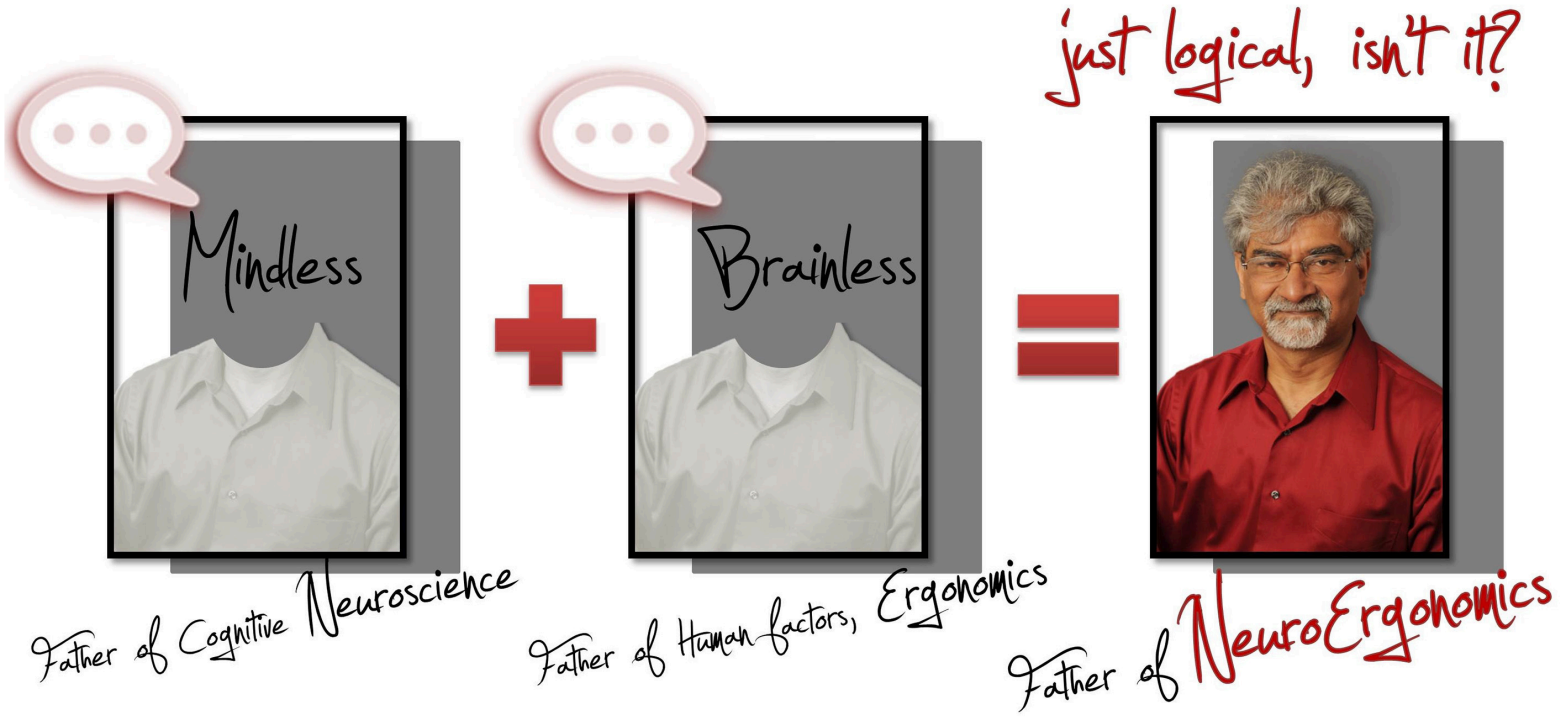
**Illustration of Raja Parasuraman as he himself wrote in an article: “One of us (Parasuraman, 1998, 2003) has therefore coined the term “neuroergonomics” to refer to the inclusion of neuroscience in Human Factors/Ergonomics (HF/E)**. Neuroergonomics can be defined as the study of brain and behavior at work. Traditionally, ergonomics has not paid much attention to neuroscience or to the results of studies of the brain mechanisms underlying human perceptual, cognitive, affective and motor processes. To paraphrase the philosopher Mario Bunge (1980), until recently psychology (and HF/E) has been “brainless,” whereas neuroscience has been “mindless.” At the same time, neuroscience and its more recent offshoot, cognitive neuroscience, have been only partially concerned with whether their findings bear any relation to human functioning in real (as opposed to laboratory) settings, with the exception of applications to clinical disorders. Neuroergonomics is a response to this twin disregard.

## Beyond the concept of mental workload and toward mental resources in neuroergonomics

MWL measurement is an important issue in the Human Factors field, as seen through its ubiquitous presence in the literature. It is well acknowledged that an accurate assessment of MWL could help to reduce human error while improving human performance. The recently founded field of *Neuroergonomics* may help to reduce the ambiguity surrounding the MWL concept by providing data on its underlying neural processes. Neuroergonomics allows for the study of the human brain structure and function with respect to behavior during physical or cognitive performances in the workplace (Mehta and Parasuraman, 2013). The main goal of this interdisciplinary field is to integrate our understanding of the neural basis of cognition in relation to technologies and settings in complex daily life tasks.

However, Neuroergonomics does not yet provide a consensual and comprehensive explanation of the MWL. Despite being a roughly defined concept, there have been some formal attempts. Generally, MWL reflects how hard one's mind is working (under- over-loaded or occupied) at any given moment or how much mental effort it will cost for brain to meet given task demands (Parasuraman, 2003). Furthermore, Parasuraman and Caggiano (2002) and Kramer and Parasuraman (2007) defined MWL as a set of mental and composite brain states that modulate human performance in different perceptual, cognitive, and/or sensorimotor skills. It is also considered as a construct used to reflect the relation between the demands of the environment (input load), the human characteristics (capacities), and the task performances (output performance). However, the notion of MWL is dissociated from performance as suggested by Ayaz et al. (2012). MWL presupposes that the consumption of true brain resources supports brain activity during work, suggesting a possible link between MWL and the key concept of *mental resources*. These two concepts can be treated by the intensity of the mental costs and be measured by the mental effort of performing tasks to predict operator performance. As stated by Cain (2007) “*As such, [MWL] is an interim measure and one that should provide insight into where increased task demands.”* Therefore, it is not possible to define MWL without also clearly characterizing mental resources.

Though it is generally admitted that mental resources are appreciable, multiple, independent, and limited (Wickens, 2008), most studies remain vague on their exact nature. One perspective is to think of mental resources as neural pathways. However, this oversimplification ignores the fact that mental resources exists in other forms. As a metaphor, an army may have efficient firepower, but without ammunition, a supply corps, and roads, it is useless. Similarly, the army of the brain has mental resources composed of neural pathways, energy supply, and irrigation (communication channel) to fuel mental effort, implemented by the mobilization of neurophysiological cellular processes in the operator's brain.

## Energy mobilization of neurovascular coupling for the operator's brain machinery

The absence of consideration of the neurophysiological mechanisms in Neuroergonomics is certainly due to the difficulty in investigating them. Yet, there are real energy mobilizations that occur within the operator's *brain machinery* across several cellular levels to meet task demand. As previously compared to a super calculator or a computer, the brain machinery supports mental processors that need substantial and constant energy requirements. But the human brain is devoid of intra-cellular capacity for energy storage in oxygen, lactate, and glucose (even if small parts of glycogen exist). Fortunately, the demand for high-metabolic energy of the brain tissue is mainly regulated by complex but adequate energetic substrate delivery via a dense and redundant network of microvessels. Hence, metabolic demands are orchestrated by the blood supply hemodynamic response.

Since the first discoveries by Roy and Sherrington (1890), it has been possible to better understand the close spatiotemporal dynamics between the electrical activity of neuronal cells and the hemodynamic phenomenon that boost the local bloodstream circulation in localized arterioles and capillaries. The intimate neurofunctional relationship that concomitantly links the metabolically active neurons with the increasing oxygenation of the blood flow near of these cells reflects the functional hyperemia and is more widely known as *neurovascular coupling* (NVC). Simply, NVC is a tight temporal association of the neuronal activity with regional cerebral blood flow delivery. Understanding the fundamental cellular mechanisms underlying NVC is necessary to measure a dimension of the local brain machinery expenditure at work. The appraisal of the energetic costs required by NVC implies the assessment of mental resources. For instance, when an operator is engaged in a task, the mobilization of the neural pathways needs a synergistic support of massive astrocyte glial cells to fuel neurons and interneurons with oxygen and nutriments furnished by close capillaries.

NVC is observable due to changes in neuronal-astroglial and microvasculature activities, which occur in several steps. First, the measurable electrical neuronal activity (spiking and postsynaptic potential activity) is accompanied by synaptic neurotransmitter release (glutamate, GABA) with a neuronal-astroglial regional cerebral metabolic rate of oxygen consumption, mainly for regional cerebral metabolic rate of glucose demand. Second, this activity induces a cascading pathway involving the production and the release of powerful vasodilator metabolites by neurons and astrocytes and drives a chemical signal up to the vascular smooth muscle and pericytes cells along the microvessels which dilate the microvasculature. Third, the microvessels dilatation significantly modulates the regional cerebral blood activity (flow, volume, and oxygenation) which greatly exceeds the neuronal-astroglial oxygen requirements, and results in a measurable overabundance of blood flow, hence, a local hyperoxygenation. Yet, the role of NVC as it contributes to the comprehension of the energy mobilization in response to mental resources is not common knowledge. The cellular measures of energy production, delivery, and utilization are crucial to understanding and interpreting NVC activity. How to clearly establish the role of NVC into the operator's brain machinery? One possible way would be to associate the level of correlates of NVC while interpreting the degree of task demand. It seems thus fairly possible that an accurate measurement of NVC, spatially and temporally and in terms of amplitude, would be a valuable neurophysiological marker for quantifying changes in brain activation. Although this statement is still reductionist (that NVC activity is proportional to operator's brain activity), this approach links the concept of human MWL and mental resources to objective neurophysiological measures for Neuroergonomics purposes.

Recent Neuroergonomics research has progressed in neurocognitive or neuroimaging-sensing instrumentation for determining operator states through the measurement of NVC activity associated with the degree of mental processes (Parasuraman and Wilson, 2008). Tremendous advances have been made toward establishing approaches for portable neuroimaging equipment and brain activation measurements to assess sensitivity to NVC in human operators acting in realistic work environments. This development is especially the case in ambulatory functional neuroimaging methods such as functional near-infrared spectroscopy (fNIRS) and electroencephalography (EEG). To date, the aforementioned non-invasive brain imaging techniques are beginning to be well-established in the Neuroergonomics community. These advantages will be even more beneficial in the future as the coupling between these methods becomes more widespread.

## Assessing neurovascular coupling with fNIRS-EEG methods: An objective neuroergonomics approach for evaluation of the operator's brain activity

Technological advances in opto- and electronic miniaturization have improved the portability and operational flexibility in brain imaging sensors, allowing for greater comprehension of the brain at work in real-world applications (aeronautics, automotive, robotics). fNIRS provides a continuous monitoring of the hemodynamic activity using near-infrared light transmitted between optodes. It infers the changes in the concentrations of oxygenated and deoxygenated hemoglobin in the cortical regions from scattering and absorption properties of light probing beneath the surface of the skull (Perrey, 2008). These two fNIRS signals have their origins in the metabolic response corresponding to a shift of oxygen consumed and the vascular response linked to a modulation of the microvasculature activity (dilatation). This hemodynamic response disrupts the regional cerebral blood flow and volume which exceeds oxygen intake (functional hyperemia) consumed by the recruited neuronal population. fNIRS responses characterize the operator's brain activity related to cerebral blood flow and cerebral tissue oxygenation changes over time (Mandrick et al., 2013; Durantin et al., 2014; Fishburn et al., 2014). Good spatial localization can be derived if a high number of optodes are used in an array, but temporal resolution is coarse by the delayed nature of the hemodynamic response to cortical activity (few seconds).

On the other hand, EEG offers a fine temporal resolution (milliseconds) thus enabling detection of brief neuronal processes, but is limited in its capacity for spatial resolution, at least in real time even though dense array EEG permits source propagation localization. EEG uses scalp electrodes to capture weak electrical current fluctuations generated by inhibitory or excitatory postsynaptic potentials of a pool of neurons firing simultaneously in response to a stimulus. The electrophysiological roots of these signals correspond to the summation of the spontaneously and synchronously recruited neuronal population that contributes to the neuronal activity of the superficial layers of the cortex. EEG waves and event-related potentials signals are particularly strong candidates for objective measures of operator's brain activity at the workplace (Parasuraman and Rizzo, 2006). In general, fNIRS and EEG are complementary as they improve on each other's measurement weaknesses in terms of information content (Fazli et al., 2012). Additionally, there is no noise cross-interference between fNIRS and EEG (light and electrical, respectively; Karanasiou, 2012). Therefore, simultaneous fNIRS-EEG signal acquisition would be suitable for assessing NVC in order to evaluate the operator's brain activity in ecological contexts (Hirshfield et al., 2009; Safaie et al., 2013).

However, it not should limit our understanding of the brain activity to only one perspective; looking at the brain at work with new tools and new eyes we could have new NVC comprehension during ecological context. Readers must note that the multimodality using fNIRS-EEG methods is a very promising approach in the investigation of where, when, and how much NVC exhibits energy mobilization during work. The spatiotemporal evolution of the functional neural connectivity and blood flow regulation through the scalp is permitted due to the recording of temporal electrical activity and spatial hemodynamic activity. Consequently, the evaluation of NVC distribution throughout the head becomes accessible. This measurement makes it possible to dynamically map the brain activity and identify the brain areas with the activated main NVC. Additionally, the assessment of the power of the electrical signal by EEG coupled with the amplitude of the hemodynamic signal by fNIRS will enable a better depiction of the intensity of the NVC, thus extrapolating the effectiveness of the metabolic effort of performing tasks. This view of the degree of extrapolated metabolic correlates as an indicator of the level of mental resources seems straightforward at first glance. However, the metabolic expenditure that fuels cognitive processes is the prerequisite for any mental resources and the assessment of operator's brain activity. The challenge now is to enhance the reliability of NVC measurement *in situ* with fNIRS-EEG methods.

## The future for neuroergonomics

It is clear that the extensive work of Parasuraman has left the scientific community in an excellent position to objectively define MWL and subsequently, mental resources, through the measurement of NVC activity. It is our opinion that NVC measurement could be achieved through the use of an efficient fNIRS-EEG coupling. In particular, there needs to be greater characterization of the energy mobilization of NVC with respect to neurophysiological mechanisms (neuronal-astroglial, metabolic and hemodynamic activity) and methods for its assessment in work settings (Parasuraman, 2011). There rests a great deal of work in Neuroergonomics before the development of a standard assessment approach of NVC with innovative neuroimaging technology for the evaluation of the operator's brain activity at work. In other words, there are still opportunities for the technological deployment of coupled hybrid devices (dry-electrodes EEG within a high density headset of fNIRS optodes). From a broader perspective, emerging devices must meet several criteria: discriminate different levels of workload; not interfere with the subject's work and environment; be accepted by the individual; be low cost with high portability; be easy to implement and to evaluate; be reproducible and reliable; and dissociate the mental workload from emotional processes (sensitivity and specificity). Theoretically, a multimodal fNIRS-EEG approach should help to investigate the interactions between different mental states and user behavior while taking into account the physiological processes. Further investigations are warranted to address newer assessments of the neurophysiological events of the operator's brain at work.

## Author contributions

Each of the authors has read and concurs with the content in the final manuscript. The first author (KM) wrote the majority of the manuscript. The other authors (ZC, MC, SP, and FD) have extensively reviewed and revised the manuscript from the first draft before giving final approval of the version to be submitted.

## Funding

This work was funded by the French Research National Agency, the French Defence Procurement Agency (ASTRID), and the AXA Research Fund.

### Conflict of interest statement

The authors declare that the research was conducted in the absence of any commercial or financial relationships that could be construed as a potential conflict of interest.
